# Heparan sulfate proteoglycans remodel SARS-CoV-2 spike conformation to allow integrin interaction and infection of endothelial cells

**DOI:** 10.3389/fcimb.2025.1552116

**Published:** 2025-04-03

**Authors:** Antonella Bugatti, Alberto Zani, Marta Bardelli, Marta Giovanetti, Cosetta Ravelli, Massimo Ciccozzi, Arnaldo Caruso, Francesca Caccuri

**Affiliations:** ^1^ Section of Microbiology, Department of Molecular and Translational Medicine, University of Brescia, Brescia, Italy; ^2^ Unit of Medical Statistics and Molecular Epidemiology, University Campus Bio-Medico of Rome, Rome, Italy; ^3^ Section of General Pathology, Department of Molecular and Translational Medicine, University of Brescia, Brescia, Italy; ^4^ Centre for Advanced Medical and Pharmaceutical Research, “George Emil Palade” University of Medicine, Pharmacy, Science and Technology, Targu Mures, Romania

**Keywords:** SARS-CoV-2, heparan sulphates, integrins, angiogenesis, von Willebrand factor, FAK-Src and Erk signaling pathway

## Abstract

SARS-CoV-2 infects ACE2-negative primary HL-mECs through the interaction of an RGD motif, included in all spike proteins, up to the Omicron BA.1 subvariant, with α_v_β_3_ integrin. Following its entry, SARS-CoV-2 remodels ECs phenotype and promotes angiogenesis in the absence of productive viral replication. Moreover, lack of spike/α_v_β_3_ interaction, occurring in Omicron BA.5 which contains the D405N mutation in the RGD motif, inhibits HL-mECs infection and dysfunction. It is worth noting that anti-spike antibodies do not impact SARS-CoV-2 entry into HL-mECs. This data highlights the fact that i) the RGD motif is not exposed in the entire spike protein and ii) the need of a cofactor favoring spike/α_v_β_3_ interaction. HSPGs are used by different viruses as receptors and coreceptors for their entry into host cells. Here, we use different approaches to scrutinize the role exerted by HSPGs in favoring SARS-CoV-2 infection of ECs. We highlight HSPGs as key molecules responsible for RGD exposure allowing its binding to the α_v_β_3_ integrin as the first step toward viral entry by endocytosis. Indeed, SPR analysis showed lack of spike/α_v_β_3_ interaction in the absence of heparin. This data was further corroborated by immunofluorescence and infectivity assays. Interestingly, the use of Heparinase III or sodium chlorate counteracts the release of proangiogenic molecules and inhibits signaling pathways induced by SARS-CoV-2 infection. Thus, HSPGs may represent a target for preventing SARS-CoV-2 infection of ECs and EC dysfunction-related COVID-19 severity.

## Introduction

Severe acute respiratory syndrome coronavirus 2 (SARS-CoV-2) infections have been mainly associated with local bronchopulmonary symptoms (Su et al., 2021; Rabaan et al., 2023). However, 20% of these infections leads to life-threatening respiratory, cardiac, renal, and cerebral injury (Odilov et al., 2021; Rabaan et al., 2023). These severe forms of disease induce an inflammatory status with a strong microvascular involvement driven by the endothelial cells (ECs) response to the infection (Canale et al., 2022). EC activation results in elevated levels of pro-inflammatory cytokines, chemokines, pro-angiogenic and anti-thrombotic factors in critical patients (Oxford et al., 2020). Indeed, it has been shown that the disruption of vascular homeostasis, secondary to EC damage, contributes to systemic proinflammatory state and multiorgan involvement observed in COVID-19 disease (Giordo et al., 2021).

Several studies demonstrated that SARS-CoV-2 infects lung ECs both *in vivo* and *in vitro* (Liu F. et al., 2021). We proved that SARS-CoV-2 infects primary human lung microvascular ECs (HL-mECs), which do not express ACE2, by using an endocytic pathway (Bugatti et al., 2022). In particular, we showed that the Arg-Gly-Asp (RGD) motif expressed in the receptor binding domain (RBD) of the spike protein, at amino acid (aa) position 403-405, is responsible for SARS-CoV-2 entry into HL-mECs through a specific α_v_β_3_ integrin interaction, giving rise to inflammatory and angiogenic responses (Bugatti et al., 2022). It is worth noting that the latest Omicron sublineages were found to differ from all previous SARS-CoV-2 VOCs for displaying a D405N mutation in their spike protein (Tuekprakhon et al., 2022; Bugatti et al., 2023), which impairs SARS-CoV-2/α_v_β_3_ integrin interaction, thus impeding EC infection and dysfunction (Bugatti et al., 2023).

Antibodies recognizing the epitope containing the RGD motif, even those evocated by BNT162b2 vaccination, demonstrated a negligible effect in neutralizing the live virus (Nitahara et al., 2021; Bugatti et al., 2022). This is due to the knowledge that the RGD motif is not exposed on the surface of the RBD neither in its “up” nor “down” conformation (Nitahara et al., 2021). Moreover, microscale accelerated molecular dynamic (MD) simulations showed that neither the RGD motif nor its microenvironment exhibit any significant conformational shift in the RBD structure able to acquire an optimal geometry for its interaction with integrins (Othman et al., 2022). These data lead to the hypothesis that, in order to expose and obtain the optimal geometry of the RGD motif in its spike, SARS-CoV-2 needs a cofactor.

Heparan sulfates (HSs) are linear polysaccharide chains found on the surface of cells and play a critical role in several biological processes (Sarrazin et al., 2011), including viral attachment (Madu et al., 2007; Cagno et al., 2019; Koganti et al., 2021). Viruses use HS interaction to improve their chances of binding to specific receptors expressed on the surface of host cells (Cagno et al., 2019). In particular, in the context of coronavirus, it has been demonstrated that SARS-CoV-2 attachment and infection involves HS-dependent enhancement of binding to ACE2 (de Haan et al., 2005; Clausen et al., 2020; Zhang et al., 2020; Liu L. et al., 2021), through conformational changes leading to RBD better exposure, thus serving as coreceptors in facilitating viral infection (Milewska et al., 2014; Clausen et al., 2020; Kim et al., 2020; Mycroft-West et al., 2020; Kalra and Kandimalla, 2021; Tandon et al., 2021; Kearns et al., 2022). These data indicate that molecules disrupting HSs/spike interaction may represent novel therapeutics against COVID-19 (Yu et al., 2021). Interestingly, it has also been suggested that SARS-CoV-2 variants evolve to be more dependent on HSs for viral attachment and infection (Kim et al., 2023).

Here, we scrutinize the role played by heparan sulfate proteoglycans (HSPGs) in promoting α_v_β_3_ integrin-mediated entry of SARS-CoV-2 into HL-mECs. We highlight HSPGs as key molecules for the first step toward viral entry by integrin-mediated endocytosis. Moreover, inhibition of HSPGs/spike interaction was sufficient to inhibit the SARS-CoV-2-sustained ECs dysfunction and related signaling pathways.

## Materials and methods

### Reagents

Recombinant trimeric Omicron BA.1 and Omicron XBB.1.5 spike proteins were purchased from BPS Bioscience (San Diego, CA, USA). The proteins were purified from HEK293 cells with a purity ≥ 90% and run at a higher molecular weight by SDS-PAGE due to glycosylation; anti-phospho-extracellular signal-regulated kinase 1/2 (pERK) and anti-total ERK_1/2_ (tERK) antibodies were purchased from Santa Cruz Biotechnology (Santa Cruz, CA, USA); anti-phospho-focal adhesion kinase (pFAK), anti phospho-Src (pSrc), anti-Glyceraldehyde-3-phosphate dehydrogenase (GAPDH) and anti-total Src (tSrc) were purchased from Cell Signaling Technology (Danvers, MA, USA); recombinant human integrin alphaV beta3 (α_v_β_3_), sodium chlorate, Heparinase III and heparin sodium salt from porcine intestinal mucosa were purchased from Merck (Darmstadt, Germany). Human α_v_β_3_ integrin antibody was purchased from Bio-Techne (Minneapolis, MN, USA).

### Cells

African green monkey kidney Vero E6 cell line was obtained from Istituto Zooprofilattico Sperimentale della Lombardia e dell’Emilia Romagna (Brescia, Italy) and maintained in Dulbecco’s Modified Eagle Medium (DMEM; Gibco, Thermo Fisher Scientific, Waltham, MA, USA) supplemented with 10% fetal bovine serum (FBS; Gibco, Thermo Fisher Scientific). Human lung microvascular endothelial cells (HL-mECs) were purchased from Lonza (Basel, Switzerland) and cultured in EGM-2 (Lonza), containing 10% FBS.

### Biacore

Surface Plasmon Resonance (SPR) measurements were conducted on a Biacore X100 (Cytiva, MN, USA) at 25°C. For the study of spike proteins/heparin interaction, biotinylated heparin was immobilized onto a SA sensor chip containing pre-immobilized streptavidin, allowing the immobilization of 186 resonance units (RU). A sensor chip pre-coated with streptavidin alone was used to evaluate nonspecific binding and for blank subtraction (Bugatti et al., 2007). Increasing concentrations of trimeric SARS-CoV-2 spike proteins belonging to Omicron BA.1 or Omicron XBB.1.5 sublineages in 10 mM HEPES, pH 7.4 containing 150 mM NaCl, 3 mM EDTA, and 0.005% surfactant P20 (HBS-EP^+^, running buffer) were injected over the heparin or streptavidin surfaces for 4 min and then washed until dissociation. After each run, the sensor chip was regenerated by injection of 2.0 M NaCl in HBS-EP^+^. Kinetic parameters of the interactions were calculated from the sensorgram overlays by using the nonlinear fitting single-site model software package BIAevaluation (version 3.2 [Cytiva]). For spike proteins/α_v_β_3_ interaction, 20 µg/ml of recombinant integrin was immobilized onto a CM5 sensor chip using standard amine-coupling chemistry allowing the immobilization of 996 resonance units, equal to 5,24 x 10^-9^ pmol/mm^2^ of α_v_β_3_. A sensor chip alone was used to evaluate nonspecific binding and for blank subtraction (Rusnati et al., 2001). Trimeric SARS-CoV-2 Omicron BA.1 or Omicron XBB.1.5 spike proteins, were diluted in running buffer and injected alone or in the presence of free heparin (10 µg/ml) over the surface for 4 min and then washed until dissociation.

### Viral infection

Infections were carried out as previously described (Bugatti et al., 2023), using the clinical SARS-CoV-2 isolates belonging to Omicron BA.1 (GISAID accession number: EPI_ISL_15700833), or Omicron XBB.1.5 (GISAID accession number: EPI_ISL_19500251) sublineages. The viruses were propagated in Vero E6 cells and the viral titer was determined by a standard plaque assay. All the experiments were performed with a single viral inoculum. Mock-infected cell cultures were obtained from uninfected cells (NI), processed exactly as the SARS-CoV-2-infected ones. All the infection experiments were carried out in a biosafety level-3 (BSL-3) laboratory at a Multiplicity of Infection (MOI) of 1. Where indicated, to inhibit the sulfation of cell-associated HS chains, HL-mECs cells were grown for 48 h in presence of sodium chlorate (50 mM). Alternatively, cells were incubated for 1 h at 37°C with Heparinase III (15 mU/ml in phosphate-buffered saline [PBS], Merck) before experimentation.

### Viral RNA extraction and qRT−PCR

RNA was extracted from infected cells using the RNeasy Plus mini kit (Qiagen, Hilden, Germany), according to the manufacturer’s instructions. RNA was eluted in 30 μl of RNase-free water and stored at -80°C until use. The qRT-PCR was carried out following previously described procedures (Bugatti et al., 2023). Each quantification was run in triplicates.

### Immunofluorescence assay

For the evaluation of spike-α_v_β_3_ localization, HL-mECs were seeded (5 x 10^4^ cells per well) on collagen-coated 8-well chamber slides (Thermo Fisher Scientific) in complete medium. After 24 h, cells were incubated at 4°C for 1 h in complete media containing 100 ng/ml of recombinant Omicron BA.1 and XBB.1.5 spike proteins. After incubation, cells were washed, fixed with 4% PFA in PBS for 10 min and saturated with 3% BSA in PBS. For staining, the cells were incubated for 1 h at 4°C with a human serum (1:1000 dilution) containing IgG against SARS-CoV-2 spike protein and with mAb against α_v_β_3_ integrin (1 µg/ml; Bio-techne) followed by Alexa Fluor 488-conjugated anti-human IgG or Alexa Fluor 594-conjugated anti-mouse IgG (Thermo Fisher Scientific). Nuclei were counterstained with DAPI (Merck). To evaluate the viral antigen in HL-mECs after infection, the cells were seeded (5 x 10^4^ cells per well) on collagen-coated 8-well chamber slides (Thermo Fisher Scientific) and infected with SARS-CoV-2 isolates belonging to Omicron BA.1 or Omicron XBB.1.5 sublineages, as previously described. After infection, cells were fixed with 4% paraformaldehyde in PBS for 10 min and stained with an anti-spike protein antibody as previously described (Bugatti et al., 2022). Cells were photographed under a Zeiss Axiovert 200 M epifluorescence microscope equipped with a Plan-Apochromat 63x/1.4 NA oil objective (Zeiss Axiovert 200M system).

### Spheroids

Spheroids were generated by mixing HL-mECs (1 x 10^5^ cells/ml) with 5 mg/mL of methylcellulose (Merck) in EGM-2 medium containing 10% FBS, making the final volume to 10 ml. The cells (100 µl/well) were then added to 96-well plates (Greiner Bio-one, Kremsmünster, Austria) and incubated at 37°C and 5% CO_2_ for 24 h. Separately, the collagen I gel solution (Rat tail, Corning, NY, USA) was maintained on ice and neutralized by adding NaOH 0.1 N and PBS 10X to a final pH of 7.4. Then, 24-well plates were coated with neutralized collagen (150 µl/well) and incubated in a humidified 5% CO_2_ incubator for 1 h at 37°C. The spheroids from 96-well plates were collected in eppendorf tubes and centrifuged at 2,000 x rpm for 10 s. When a clear pellet was distinguished, the supernatant was removed, and the pellet was kept in a volume of about 150 µl collagen I-neutralized solution. Each collagen-spheroid mixture was rapidly added to the pre-coated 24-well plates (200 µl/well) and incubated for 1 h. After 1 h, 500 µl of EGM-2 containing 10% FBS, was added to the wells to completely cover the surface and plates were further incubated for 24 h. Where indicated, HL-mECs cells were grown for 48 h in presence of sodium chlorate or alternatively, ECs were incubated for 1 h at 37°C with Heparinase III before experimentation. Sprouting occurred from the spheroid core, photographed with a Hitachi KP-D50 camera (Hitachi Ltd., Tokyo, Japan), and the number of sprouts was counted with the spheroids of similar sizes from three different wells of the plate.

### Microarray analysis

Supernatants from infected treated and not treated HL-mECs were collected at 3 days post infection (p.i.), clarified and analyzed for the expression of 55 different angiogenesis-related proteins by Human Angiogenesis Array Kit (Proteome Profiler, R&D systems, Minneapolis, USA) according to the manufacturer’s instructions.

### Fluorescent vWF expression and quantification

HL-mECs were nucleofected with a mCherry-vWF expressing plasmid (Bugatti et al., 2020) by using the Amaxa Nucleofector Technology (Lonza). Twenty-four h after nucleofection, HL-mECs were infected with SARS-CoV-2 belonging to Omicron BA.1 lineage and the fluorescence was analyzed at different time points (1, 3, 6 and 24 h p.i.). When reported, HL-mECs were treated with sodium chlorate and Heparinase III, for 48 h or 1 h respectively, before the SARS-CoV-2 infection. Nuclei were counterstained with DAPI (Merck). Fluorescence was analyzed using a Zeiss Axiovert 200 M epifluorescence microscope equipped with a Plan-Apochromat 63x/1.4 NA oil objective (Zeiss Axiovert 200M system). The number of puncta per cell was quantified using Image J software (Fiji, NIH, Bethesda, USA), by counting vWF-positive puncta in 20 cells/experiment. Error bars represent the standard deviation calculated as the mean of 3 independent experiments with similar results.

### Human vWF ELISA array

Supernatants from mCherry-vWF expressing plasmid nucleofected and SARS-CoV-2 infected HL-mECs were collected at different time points (1, 3, 6 and 24 h p.i.), clarified and analyzed for the level of secreted vWF by Human vWF ELISA Kit (Merck) according to the manufacturer’s instructions.

### Signaling pathways activation assay

HL-mECs were infected with SARS-CoV-2 belonging to Omicron BA.1 or XBB.1.5 sublineages as previously described and 1 h p.i. the cells were lysed in 50 mM Tris-HCl pH 7.4 containing 150 mM NaCl, 1% Triton X-100, 1 mM Phenylmethylsulfonyl fluoride (PMSF) and Protease Inhibitor Cocktail (Merck) and centrifuged. Twenty µg of total proteins were analyzed on SDS-12% PAGE followed by Western blotting with anti-pFAK, anti-p-Src and anti-pERK antibodies. Equal loading of the lanes was confirmed by immunoblotting with anti-tSrc, anti-tERK or anti-GAPDH antibodies. The intensity of the pSrc, pERK and pFAk signal was quantified and normalized to the intensity of the corresponding tSrc, tERK and GAPDH band using Image J software.

### Monitoring of recent SARS-CoV-2 strains with reverse mutation S405D and phylogenetic analysis

A total of 1083 SARS-CoV-2 whole-genome sequences, carrying all four mutations of interest (D405D, D405N, N405S), as well as recent strains with the reverse mutation S405D, selected based on the availability of comprehensive associated metadata, were analyzed in this study. Each sequence was aligned using the ViralMSA tool (Moshiri, 2021). For phylogenetic analysis, we employed IQ-TREE2 (Minh et al., 2020) with a maximum likelihood approach. To provide a temporal framework, we then used TreeTime (Sagulenko et al., 2018) to transform the raw maximum likelihood tree into a dated tree, applying a constant mean substitution rate of 8.0 x 10^-4^ nucleotide substitutions per site per year. This temporal calibration, after excluding outlier sequences, allowed us to place each sample in a chronological context, enhancing our understanding of the timing and dynamics of viral spread.

### Statistical analysis

Data were analyzed for statistical significance using the Student’s two-tailed t-test or one-way ANOVA when appropriate. Bonferroni post-test was used to compare data. Differences were considered significant when P < 0.05. Statistical tests were performed using Prism 8 software (GraphPad Software, La Jolla, CA, USA).

## Results

### HSPGs mediate spike/α_v_β_3_ interaction

**Figure 1:**
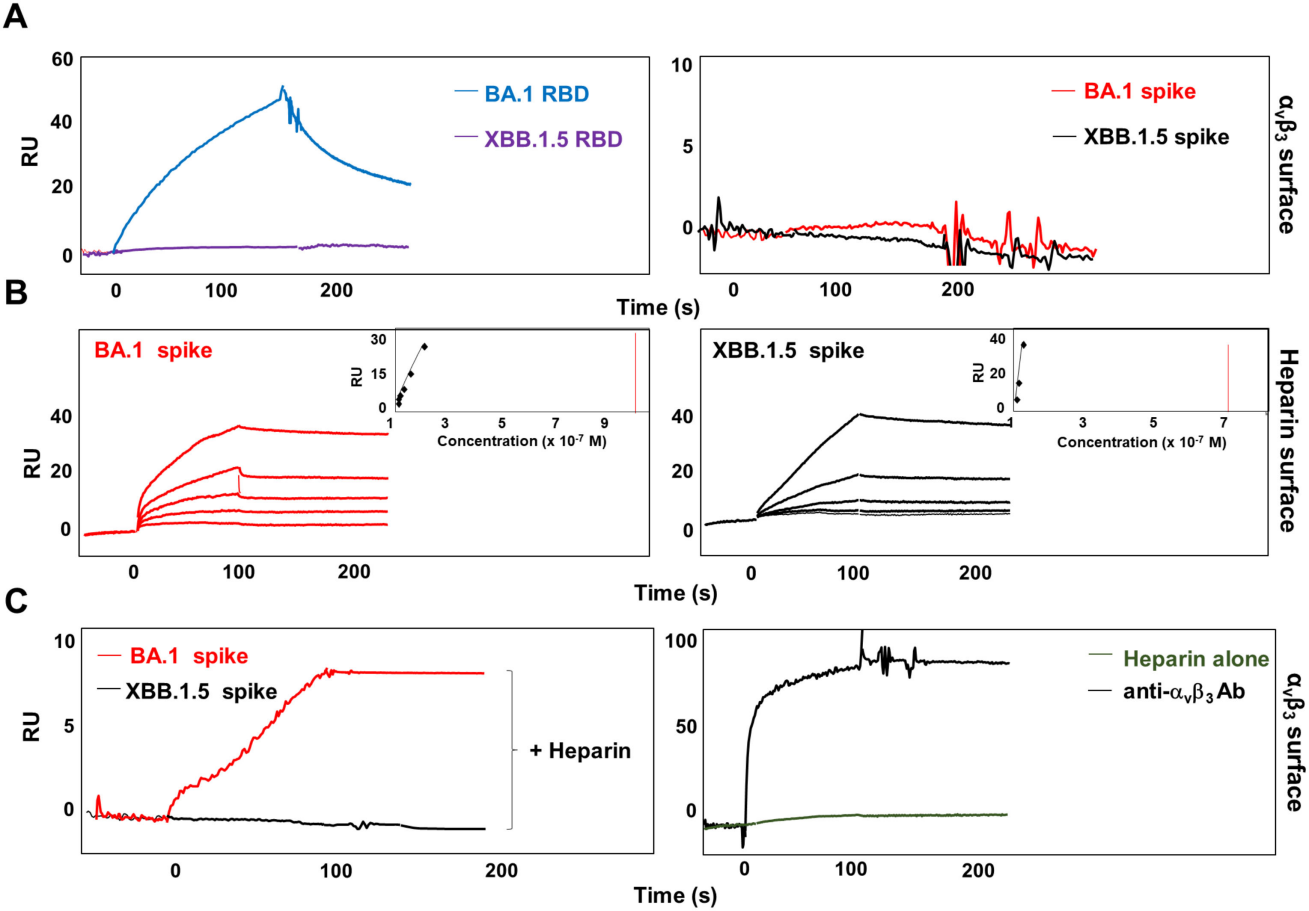
Heparin mediates SARS-CoV-2 Omicron BA.1 interaction with α_v_β_3_ integrin. **(A)** BA.1 (blue line) and XBB.1.5 (purple line) RBDs or BA.1 (red line) and XBB.1.5 (black line) spike proteins were injected onto α_v_β_3_ surface at a single concentration. **(B)** Sensogram overlay showing the binding of increasing amounts of BA.1 (range from 6.25 to 100 nM, left panel) and XBB.1.5 (range from 1.56 to 25 nM, right panel) spike proteins to heparin surface. The response, in resonance units (RU), was recorded as a function of time. Inset of left and right panels: equilibrium binding data generate the saturation curve and scatchard plot regression. **(C)** BA.1 (red line, left panel) and XBB.1.5 (black line, left panel) spikes were co-injected with heparin onto the α_v_β_3_ surface. Antibody against α_v_β_3_ (black line, right panel) and soluble heparin alone (green line, right panel) were injected as control.

The RGD motif is not exposed on the surface of the RBD domain of the entire spike protein (Nitahara et al., 2021). It is worth noting that the optimal RBD conformation for ACE2 binding is promoted by HSPGs/spike interaction (Clausen et al., 2020). Previously, we demonstrated that the RBD domain of Omicron BA.1 endowing the RGD motif, but not the one belonging to Omicron BA.5 carrying the D405N mutation, directly interacts with α_v_β_3_ (Bugatti et al., 2023). To assess whether a correct three-dimensional configuration of the entire trimeric spike protein of SARS-CoV-2 is required to bind the α_v_β_3_ integrin, the latter was immobilized on a CM5 sensor chip. Two recombinant trimeric SARS-CoV-2 spike proteins were used; one belonging to Omicron BA.1, displaying the RGD motif, (BA.1 spike) and the other belonging to Omicron XBB.1.5, displaying the mutated RGN domain, (XBB.1.5 spike). The RBD domains of Omicron BA.1 and XBB.1.5 (BA.1 RBD and XBB.1.5 RBD, respectively) were used as control of interaction. Interestingly, as shown in **Figure 1A**, right panel, the trimeric SARS-CoV-2 spike protein belonging to the Omicron BA.1 lineage, differently from its RBD counterpart (**Figure 1A**, left panel), did not show any interaction with immobilized α_v_β_3_ integrin. As expected, trimeric Omicron XBB.1.5 spike protein (**Figure 1A**, right panel), as well as its RBD domain (**Figure 1A**, left panel), is not capable of binding α_v_β_3_ integrin. Based on this evidence, we hypothesized that to convert the RBD from inaccessible to accessible conformation and to expose the RGD motif, SARS-CoV-2 spike protein needs a cofactor. Past work shows that HSPGs, abundant components of the extracellular matrix (ECM) (Hassan et al., 2021), binds to spike proteins and increase the number of spike RBDs in the up conformation (Zhang et al., 2024). Thus, we speculate that HSPGs may act as cofactors favoring spike/α_v_β_3_ interaction. To test this hypothesis, we used an SPR model that mimics the binding of proteins to cell surface-associated HSPGs immobilizing biotinylated heparin on the sensor chip. As shown in **Figure 1B**, both BA.1 and XBB.1.5 spike proteins interact with immobilized heparin (left panel and right panel, respectively), in a dose-dependent manner. The sensorgram overlay allowed the calculation of association (*k*_on_) and dissociation (*k*_off_) rates and of *Kd* (as *k*_off_/*k*_on_ ratio) value. Kinetic parameters of spike proteins/heparin interaction are reported in **Table 1**. Equilibrium binding data were used to generate the saturation curve and scatchard plot regression (inset panels in **Figure 1B**) and to calculate a *Kd* value independent from kinetic parameters (**Table 1**). This evidence prompted us to deeply scrutinize whether spike/heparin interaction could induce the exposure of the RGD motif thus allowing its interaction with α_v_β_3_ integrin. Interestingly, as shown in **Figure 1C**, BA.1 spike protein (left panel, red line) acquired the capacity to bind the immobilized α_v_β_3_ when injected in the presence of soluble heparin. On the contrary, no interaction was observed between immobilized α_v_β_3_ and XBB.1.5 spike (left panel, black line), carrying the mutated RGD motif, in the presence of heparin (**Figure 1C**). Antibody against α_v_β_3_ (black line) was used as positive control of surface specificity while soluble heparin alone (green line) was used as negative control of interaction (**Figure 1C**, right panel). This result confirmed the hypothesis that HSPGs act as a cofactor that promote the RBD open conformation and the exposure of the RGD motif in SARS-CoV-2 Omicron BA.1 spike protein allowing its interaction with α_v_β_3_ integrin.

**Table 1:** Kinetic and affinity parameters of SARS-CoV-2 Omicron BA.1 and XBB.1.5 spike proteins on immobilized Heparin.

	K_on_ (1/Ms)	K_off_ (1/s)	Kd (M)	Kd_eq_ (M)
**Omicron BA.1**	3.137 x 10^5^	0.5616	1.790 x 10^-6^	8.807 x 10^-7^
**Omicron XBB.1.5**	2.425 x 10^5^	0.02681	1.105 x 10^-7^	6.022 x 10^-7^

**Figure 2:**
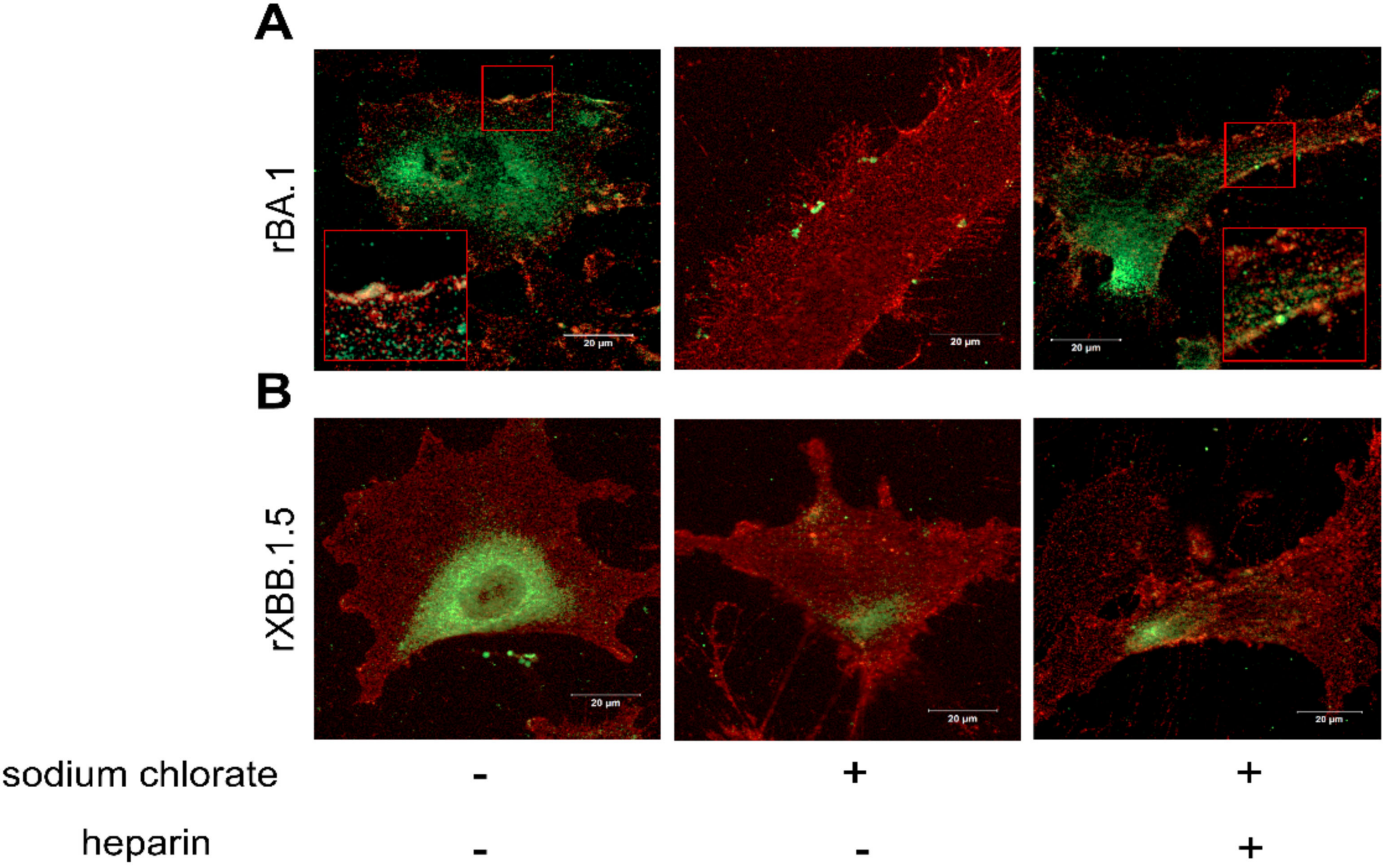
HSPGs mediate SARS-CoV-2 Omicron BA.1 interaction with α_v_β_3_ integrin on HL-mECs. HL-mECs were incubated with recombinant Omicron SARS-CoV-2 BA.1 (**A,** left panel) or XBB.1.5 (**B,** left panel) spike proteins (rBA.1 and rXBB.1.5, respectively) for 2 h at 4°C, then washed and fixed with 4% PFA in PBS. For staining, cells were incubated for 1 h at 4°C with a human serum containing IgG to SARS-CoV-2 or anti-α_v_β_3_ antibody followed by Alexa Fluor 488-conjugated anti-human IgG or Alexa Fluor 594-conjugated anti-mouse IgG, respectively. Nuclei were counterstained with DAPI. Images display SARS-CoV-2 spike signal in green, α_v_β_3_ signal in red and cell nuclei in blue. HL-mECs were pretreated with sodium chlorate, incubated with rBA.1 (**A,** middle panel) and rXBB.1.5 (**B,** middle panel) and decorated as described above. After all, pretreated sodium chlorate-HL-mECs were incubated with a mix solution of rBA.1 (**A,** right panel) or rXBB.1.5 (**B,** right panel) spike proteins and soluble heparin (preincubation at 37°C for 1 h) and decorated as above. Scale bar, 20 µm.

To further confirm the SPR experiments, an immunofluorescence (IF) assay was performed. For this purpose, HL-mECs were incubated for 1 h at 4°C with recombinant SARS-CoV-2 Omicron BA.1 and Omicron XBB.1.5 spike proteins and then fixed before being stained with antibodies against the spike proteins (green signal) and α_v_β_3_ integrin (red signal). As shown in **Figure 2A**, BA.1 spike protein localized on the surface and along the contour of the cell at the integrin signal (left panel), whereas the XBB.1.5 spike protein localized on the surface of the HL-mECs only, and was not localize along the integrin signal (**Figure 2B**, left panel). This result demonstrates that both BA.1 and XBB.1.5 proteins bind to HSPGs, but only the BA.1 spike protein is also able to interact with α_v_β_3_ integrin via its RGD motif. When HL-mECs were pretreated with sodium chlorate, no surface signal was observed for both BA.1 and XBB.1.5 spike proteins (**Figures 2A, B**, middle panel, respectively), thus confirming that HSPGs are involved in the attachment of SARS-CoV-2 spikes to the ECs. To assess whether soluble heparin was able to rescue the ability of the spike proteins to bind α_v_β_3_ integrin in HL-mECs pretreated with sodium chlorate, BA.1 and XBB.1.5 spike proteins were pre-incubated with soluble heparin (10 µg/ml) at 37°C for 1 h and then incubated for 1 h at 4°C on HL-mECs. As shown in **Figure 2A** (right panel), the localization of BA.1 spike protein was overlapping with that observed in untreated HL-mECs, indicating an interaction between BA.1 and α_v_β_3_ integrin. As expected, XBB.1.5 spike protein was not observed on the surface of HL-mECs not expressing HSPGs (**Figure 2B**, right panel). Taken together, our data confirmed the ability of heparin/HSPGs to promote the exposure of the RGD motif in the BA.1 spike protein, allowing its subsequent binding to the α_v_β_3_ integrin.

### HSPGs mediate Omicron BA.1-HL-mECs infection

SPR and IF experiments demonstrated that the trimeric spike/integrin interaction depends on the presence of HSPGs. To verify *in vitro* that HSPGs are indispensable to promote SARS-CoV-2 attachment and infection of HL-mECs, Heparinase III or sodium chlorate pretreated-HL-mECs were infected with 1 MOI of authentic SARS-CoV-2 belonging to Omicron BA.1 lineage. SARS-CoV-2 Omicron XBB.1.5 lineage, carrying the D405N mutation, was used as a negative control of infection.

**Figure 3:**
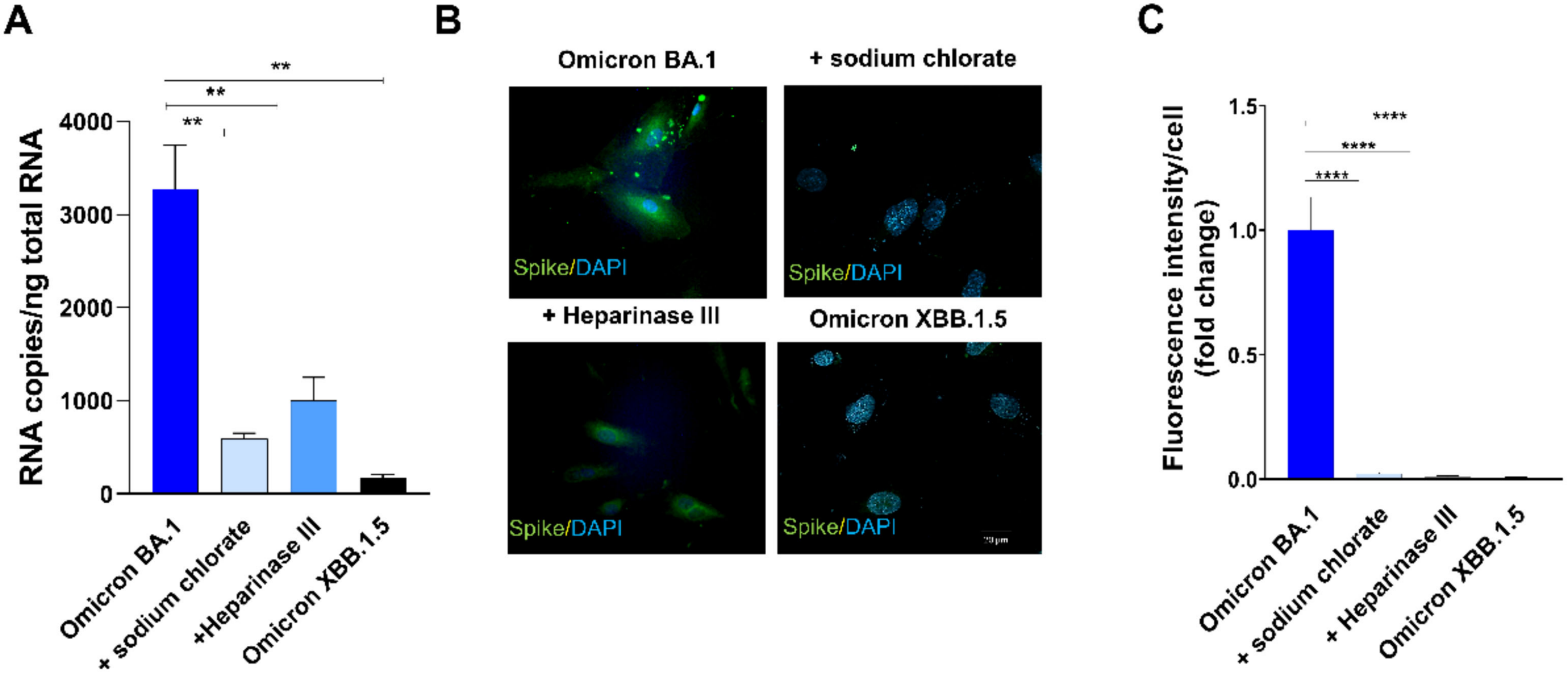
HSPGs are indispensable to mediate Omicron BA.1-HL-mECs infection. HL-mECs were pretreated with Heparinase III and sodium chlorate for 1 h and 48 h at 37°C, respectively. After treatment, the cells were infected with Omicron BA.1 and Omicron XBB.1.5 at a MOI of 1 for 1 h at 37°C, then washed and cultured until day 1 p.i. **(A)** The graph shows quantification of SARS-CoV-2 genomes at the intracellular level by qRT-PCR. At least three replicates were performed. Data are representative of two independent experiments with similar results. Statistical analysis was performed by one-way ANOVA (**p = 0.0023). **(B)** HL-mECs were fixed, permeabilized, and saturated with BSA. For staining, cells were incubated for 1 h at 37°C with a human serum containing IgG to SARS-CoV-2 and followed by Alexa Fluor 488-conjugated anti-human IgG. Nuclei were counterstained with DAPI. Images display SARS-CoV-2 signal in green and cell nuclei in blue (scale bar, 20 µm). **(C)** Spike-related fluorescent area was quantified in 20-30 Omicron BA.1-infected cells pretreated or not with sodium chlorate or Heparinase III and subtracted from not-infected cells fluorescence signal. Decrease of spike-related fluorescence in pretreated cells with sodium chlorate or Heparinase III Omicron BA.1 infected-cells was related to Omicron BA.1 signal and was expressed as fold change. Statistical analysis was performed by unpaired t-test (****p < 0.0001).

Twenty-four h p.i., SARS-CoV-2 viral genome and protein expression were evaluated by quantitative real-time PCR and indirect IF assay, respectively. As shown in **Figure 3A**, quantification of intracellular SARS-CoV-2 RNA showed a significant reduction of viral RNA in Heparinase III- and sodium chlorate-pretreated HL-mECs as compared to SARS-CoV-2 Omicron BA.1-infected ones. As expected, Omicron XBB.1.5 did not infect HL-mECs. Similar results were obtained by evaluating viral protein expression in HL-mECs by IF. As shown in **Figure 3B**, the presence of SARS-CoV-2 spike protein was observed in Omicron BA.1-infected HL-mECs, whereas viral protein expression was not observed in Heparinase III and sodium chlorate pretreated-cells, as well as in Omicron XBB.1.5 infected-ones. Quantification of the spike-related fluorescence in Omicron BA.1-infected cells pretreated with HSPGs inhibitors confirmed the absence of viral proteins (**Figure 3C**). Taken together, these findings demonstrate that the binding of SARS-CoV-2 spike protein to HSPGs on the cell surface is indispensable to expose the RGD motif thus allowing its interaction with ECs and promoting virus infection.

### HSPGs are favor Omicron BA.1-induced angiogenic phenotype

**Figure 4:**
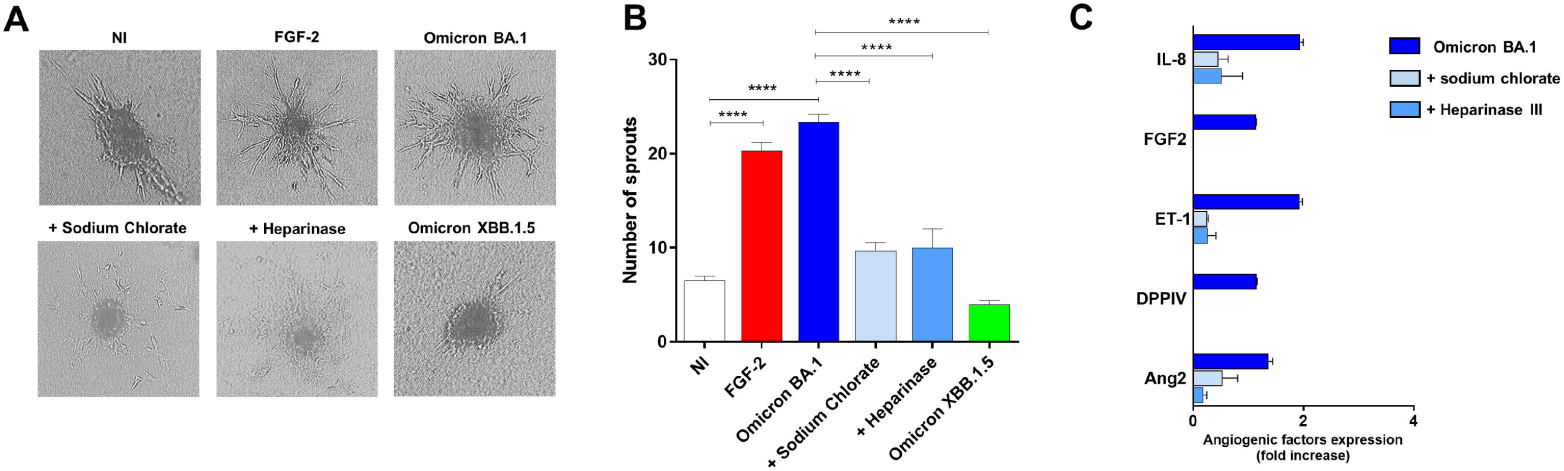
HSPGs inhibitors-treated HL-mECs do not acquire an angiogenic phenotype and do not release angiogenic factors upon Omicron BA.1 infection. HL-mECs were not-infected (NI) or infected with SARS-CoV-2 belonging to Omicron BA.1 or XBB.1.5 sublineages at MOI 1, for 1 h at 37°C and then washed and cultured until day 3 p.i. When indicated HL-mECs were pretreated with sodium chlorate (50 mM) or Heparinase III (5 mU/ml). **(A)** Sprouting of spheroids generated with NI, Omicron BA.1, and HSPGs inhibitors pretreated-HL-mECs. Pictures are representative of one out of three independent experiments with similar results (scale bar, 10 μm). FGF-2 was used as a positive control. **(B)** Values reported in the graph are the mean ± SD of one representative experiment out of three independent experiments with similar results performed in triplicate. Statistical analysis was performed by one-way ANOVA and Bonferroni’s post-test was used to compare data (****p < 0.0001). **(C)** Sodium chlorate and Heparinase III pretreated- or not HL-mECs, were infected with SARS-CoV-2 Omicron BA.1 lineage at MOI 1, for 1 h at 37°C and then washed and cultured until day 3 p.i. Clarified supernatants were evaluated for the presence of angiogenic molecules by a human proteome array. The results are expressed as mean values ± SD of duplicates given as fold increase as compared to NI cells. Data are representative of one out of two independent experiments with similar results.

To scrutinize the role of HSPGs in Omicron BA.1-induced angiogenesis, we took advantage of the spheroid assay, a three-dimensional (3D) cell model that mimics *in vivo* sprouting angiogenesis (Caccuri et al., 2021). To this end, 3 days p.i., HL-mECs infected with SARS-CoV-2 belonging to Omicron BA.1 and Omicron XBB.1.5 sublineages, pretreated or not with sodium chlorate or Heparinase III, were collected to generate spheroids (Chiodelli et al., 2011). As shown in **Figure 4A**, after 24 h of observation, non-infected spheroids (NI) did not develop any sprout while in SARS-CoV-2 Omicron BA.1-infected spheroids (Omicron BA.1) a consistent outgrowth of sprouts was observed. The effect of Omicron BA.1 infection on sprouting angiogenesis was found to be superimposable to that observed upon FGF-2 treatment, representing the positive control. Interestingly, spheroids generated by using HSPGs inhibitors-pretreated cells infected with Omicron BA.1, showed a dramatic reduction of vessel outgrowth (**Figure 4A**). Once again, the Omicron XBB.1.5 virus, carrying the RGN motif, was not able to gain access into ECs and to induce pro-angiogenic activity.

In order to understand whether HSPGs inhibition was impacting ECs pro-angiogenic functions upon Omicron BA.1 infection, we analyzed the infected-HL-mECs secretome at day 3 p.i., by using a human angiogenic array. As expected, SARS-CoV-2 Omicron BA.1 triggered the secretion of different pro-angiogenic molecules, in particular Interleukin-8 (IL-8), Fibroblast growth factor-2 (FGF-2), Angiopoietin-2 (Ang2), Endothelin-1 (ET-1) and dipeptidyl-peptidase IV (DPPIV) (**Figure 4B**, dark blue bars). The release of these factors was strongly inhibited by sodium chlorate or Heparinase III pretreatment of HL-mECs (**Figure 4B**, light blue and azul bars, respectively) confirming that lack of HSPGs on HL-mECs inhibits viral infection and the release of angiogenic factors in the microenvironment.

### SARS-CoV-2 induces accumulation and degradation of von Willebrand factor into ECs

**Figure 5:**
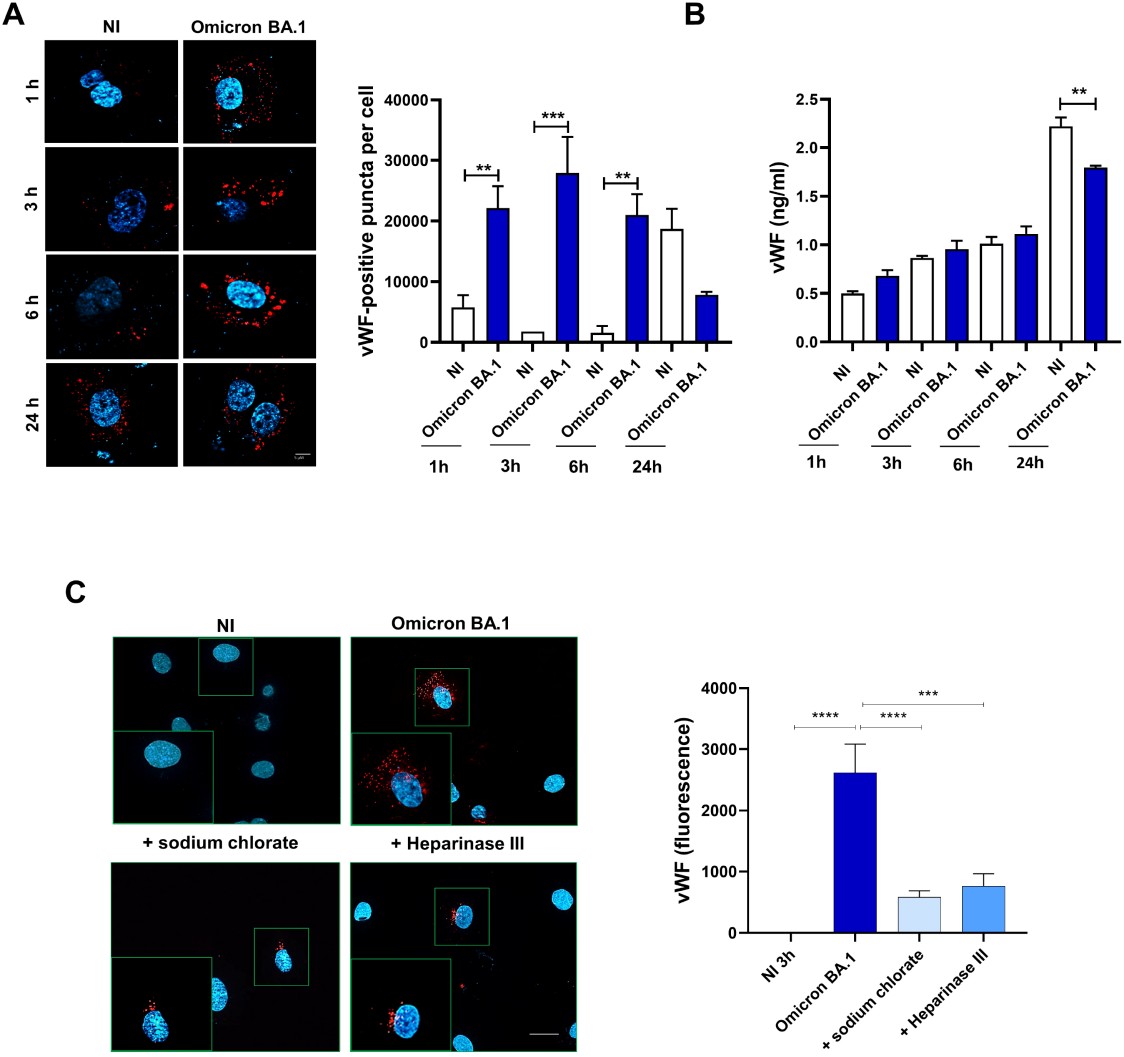
SARS-CoV-2 induces vWF accumulation in WPBs and subsequently degradation in ECs. HL-mECs were nucleofected with a mCherry-vWF-expressing plasmid and 24 h after nucleofection, cells were infected with Omicron BA.1 virus and analyzed at different time points (1, 3, 6 and 24 h). (**A,** left panel) The images display vWF signals in red and cell nuclei in blue (scale bar, 10 μm). Red-positive punctate structures were counted in order to quantify the levels of WPBs. (**A,** right panel) Values reported for vWF positive structures are the mean ± SD of 3 independent experiments with similar results. **(B)** Amount of vWF in SARS-CoV-2 infected HL-mECs supernatants are the mean ± SD of 3 independent experiments with similar results. Statistical analysis was performed by one-way ANOVA, and the Bonferroni post-test was used to compare data (**p = 0.022, ***p = 0.0008). **(C)** Infected HL-mECs pretreated or not with HSPGs inhibitors were analyzed at 3 h p.i. Values reported for vWF positive structures are the mean ± SD of 3 independent experiments with similar results. Statistical analysis was performed by one-way ANOVA, and the Bonferroni post-test was used to compare data (***p = 0.0008, ****p < 0.0001).

Some viruses, such as Kaposi sarcoma-associated herpesvirus (KSHV) induce the release of IL-8, Ang2, ET-1, and von Willebrand factor (vWF) in ECs (Ye et al., 2013). Furthermore, it has been shown that SARS-CoV-2 spike protein induces the release of vWF from ECs (Li et al., 2022). Interestingly, we found the presence of IL-8, Ang2 and ET-1 in SARS-CoV-2-infected HL-mECs secretome. This finding led us to investigate whether the authentic virus was able to induce the storage and the release of vWF which is known to exert multiple vascular roles in ECs including angiogenesis (Starke et al., 2011). To this aim, a mCherry-vWF-expressing plasmid was used to nucleofect ECs and to monitor vWF accumulation in Weibel Palade Bodies (WPBs) in Omicron BA.1 infected-HL-mECs at different time points (1, 3, 6, and 24 h p.i.). As shown in **Figure 5A**, upon SARS-CoV-2 infection, Omicron BA.1 infected-HL-mECs (Omicron BA.1) showed an increase of vWF accumulation until 6 h p.i. and a decrease at 24 h p.i., as compared to NI cells. To evaluate if the amount of vWF observed in infected HL-mECs was secreted in the extracellular environment or degraded upon time, we quantified the amount of vWF in cell supernatants by an ELISA array. As shown in **Figure 5B**, there was no difference in the quantity of vWF found in infected or NI cells at 1, 3 and 6 h p.i. Interestingly at 24 h p.i., a significant amount of secreted vWF was observed in the supernatants of NI cells as compared to the infected ones. These data suggest that the accumulated vWF, observed in infected-HL-mECs, is not released into the extracellular environment, but it is likely to be degraded within cells. As expected, in NI cells after its accumulation vWf is released within 24 h of observation (**Figure 5B**). vWF decrease observed in our results could be related to the strong expression of the angiogenic factors previously described in HL-mECs secretome, as already shown for Ang2 (Randi et al., 2018).

To further scrutinize the direct involvement of virus entry in vWF accumulation and degradation, we pretreated HL-mECs with HSPGs inhibitors before SARS-CoV-2 infection. As shown in **Figure 5C**, pretreatment with sodium chlorate or Heparinase III strongly inhibited (78% and 71%, respectively) vWF accumulation into infected HL-mECs demonstrating that the block of viral binding to HSPGs and subsequently to integrin, directly correlates with vWF presence in WPBs. Taken together, our data demonstrate that cytoplasmic vWF increase upon SARS-CoV-2 infection is specific and depends on the virus entry.

### SARS-CoV-2 promotes angiogenesis through the activation of FAK/Src/ERK signaling pathways

**Figure 6:**
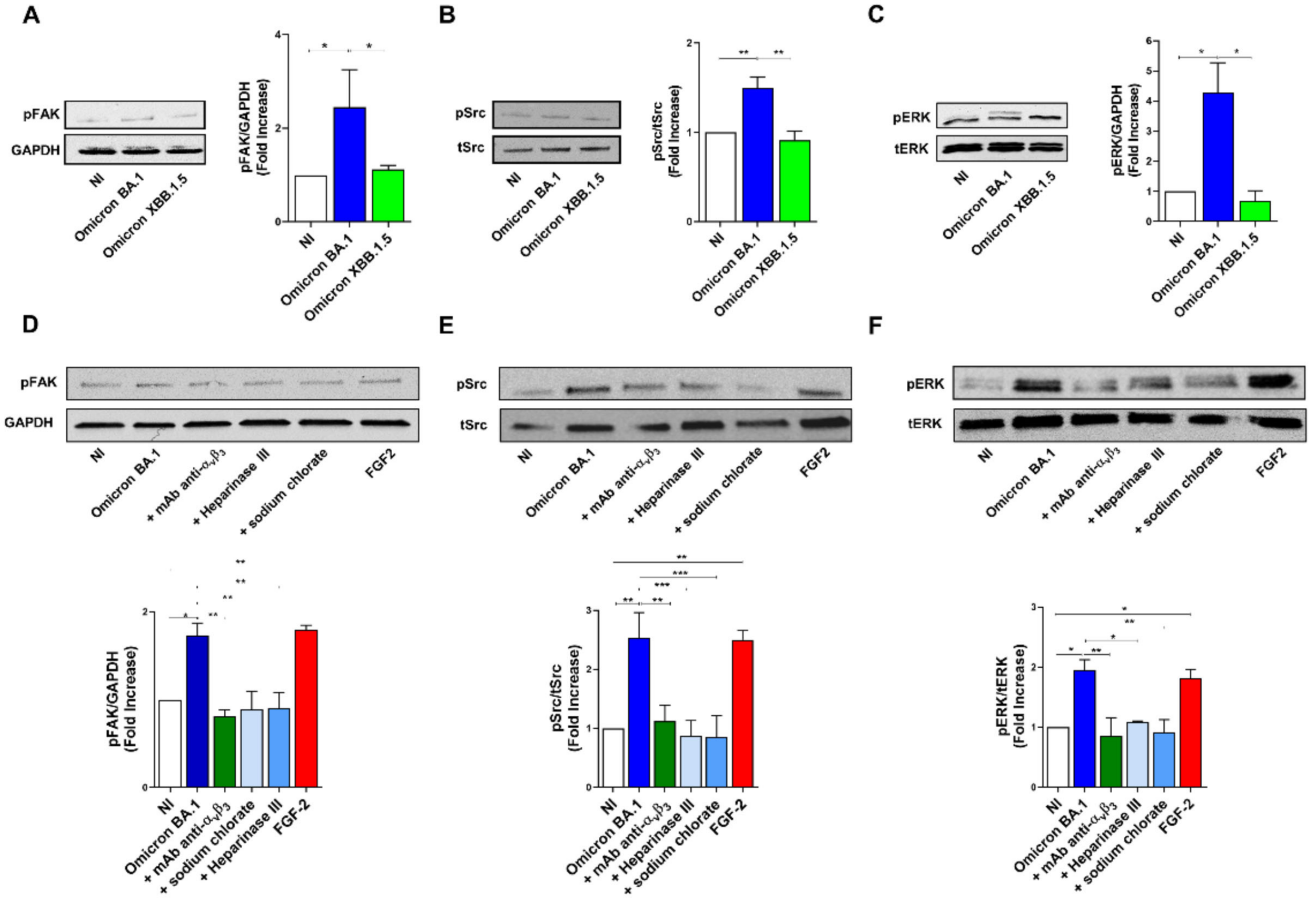
SARS-CoV-2 Omicron BA.1 induces FAK/Src and ERK signaling after α_v_β_3_ interaction. HL-mECs were not infected (NI) or infected with Omicron SARS-CoV-2 belonging to BA.1 and only XBB.1.5 sublineages (Omicron BA.1 and Omicron XBB.1.5, respectively) at a MOI of 1 for 1 h at 37°C. After 1 h p.i., cell lysates were analyzed by western blotting with anti-pFAK, anti-pSRC and anti-pERK antibodies (**A-C**, respectively). Blots are representative of three independent experiments with similar results. Quantification was carried out by densitometric analysis and plotting of the pFAK, pSrc and pERK normalized on GAPDH, tSrc or tERK as indicated in the graph. Values reported are the means ± the SD of three independent experiments. Statistical analysis was performed by one-way ANOVA, and the Bonferroni post-test was used to compare data (*p = 0.05, **p = 0.022, ***p = 0.0008). When indicated HL-mECs were pretreated with a mAb against α_v_β_3_ integrin (mAb anti- α_v_β_3_) or HSPGs inhibitors and analyzed for FAK/Src and ERK phosphorylation status **(D-F)**.

The binding to integrin receptors activates tyrosine kinase signaling pathways, leading to phosphorylation of FAK, and Src (Ye et al., 2013). In particular, α_v_β_3_ integrin promotes signaling events necessary for vascular cell survival, thereby facilitating the induction and/or maintenance of the angiogenic phenotype (Eliceiri et al., 1998). This knowledge prompted us to investigate the ability of SARS-CoV-2 to induce a productive cross-talk between spike and α_v_β_3_ integrin to trigger FAK/Src signaling in infected HL-mECs. To this aim, the cells were infected with Omicron BA.1 and Omicron XBB.1.5, as previously described. At 1 h p.i., HL-mECs were lysed and 20 µg of total proteins were analyzed by western blotting with anti-p-FAK and anti-p-Src antibodies. As shown in **Figures 6A, B**, Omicron BA.1 induced the phosphorylation of FAK and Src (2.3 ± 0.7 and 1.8 ± 0.5-fold increase, respectively). On the contrary, Omicron XBB.1.5, carrying the D405N mutation, does not activate FAK/Src signaling pathways (**Figures 6A, B**), thus supporting the direct role exerted by α_v_β_3_ in promoting FAK/Src phosphorylation in ECs. This data also indicates that spike/HSPGs interaction is not sufficient to direct HL-mECs toward a pro-angiogenic phenotype.

To further confirm the role played spike/α_v_β_3_ interaction in inducing an angiogenic signaling activation, HL-mECs were pretreated with a neutralizing monoclonal antibody against α_v_β_3_ integrin (mAb anti-α_v_β_3_), Heparinase III or sodium chlorate, then infected with Omicron BA.1 and analyzed for FAK/Src signaling activation. As shown in **Figures 6D, E**, Omicron BA.1 was able to induce an increase of FAK and Src phosphorylation as compared to NI-HL-mECs while mAb anti-α_v_β_3_, Heparinase III and sodium chlorate treatment induced a significant inhibition of FAK (54%, 48% and 48% respectively) and pSrc (56%, 66% and 65%, respectively) activation.

Since pERK signal transduction pathway in ECs is required for angiogenesis and correlates with FAK/Src activation (Murphy et al., 2006), we investigated ERK phosphorylation upon SARS-CoV-2 infection. To this end, HL-mEC were infected with SARS-CoV-2 Omicron BA.1 or Omicron XBB.1.5 in the presence or absence of the mAb anti-α_v_β_3_ or HSPGs inhibitors. As shown in **Figure 6C**, Omicron BA.1, but not Omicron XBB.1.5, was able to induce a strong increase (3.5 ± 1.5-fold increase) in ERK phosphorylation as compared to NI cells. As expected, ERK activation was inhibited when HL-mECs were pretreated with the mAb anti-α_v_β_3_ or HPSGs inhibitors, in particular sodium chlorate treatment inhibited SARS-CoV-2-ERK phosphorylation of about 53%, while mAb anti-α_v_β_3_ and Heparinase III treated-HL-mECs showed an inhibition of ERK activation equal to 44% and 56%, respectively (**Figure 6F**). Our data highlight that the docking of spike protein to HSPGs allows its interaction with α_v_β_3_, thus promoting the angiogenic signaling pathway in SARS-CoV-2-infected HL-mECs.

### Recent SARS-CoV-2 strains carry the reverse mutation S405D

**Figure 7:**
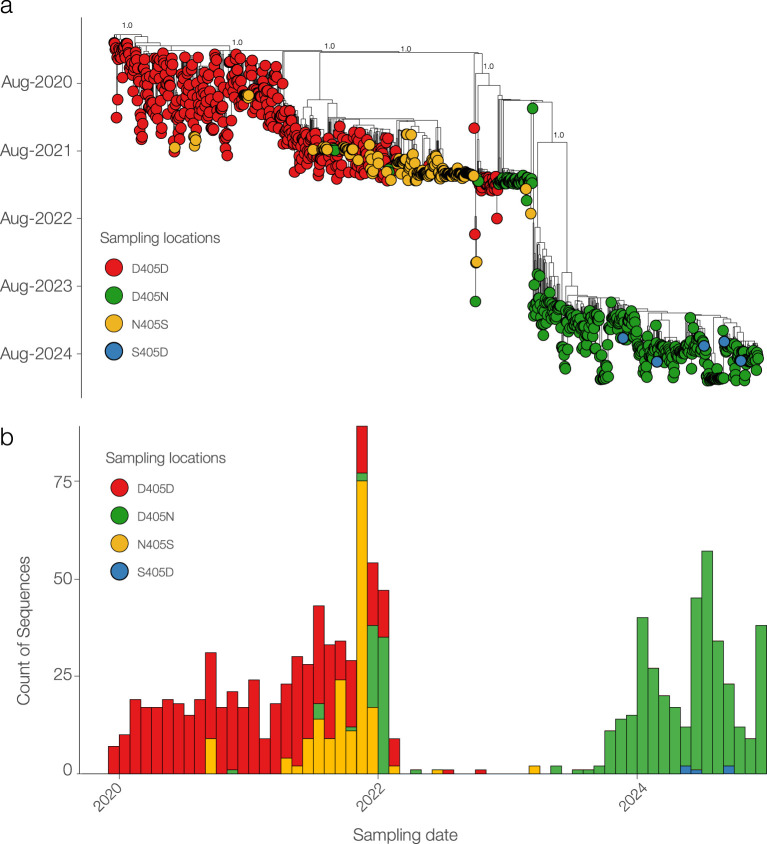
Phylogenetic and temporal analysis of SARS-CoV-2 sequences carrying D405D and its mutations. **(A)** Maximum likelihood phylogenetic tree showing the distribution of sequences with D405D (red) and its alternative substitutions: D405N (green), N405S (yellow), and S405D (blue). **(B)** Temporal distribution of SARS-CoV-2 sequences by substitution type.

Phylogenetic analysis, based on 1,083 SARS-CoV-2 whole-genome sequences, revealed distinct evolutionary patterns among the four mutations of interest: D405D, D405N, N405S, and the reverse mutation S405D (**Figure 7A**). D405D was the predominant substitution from 2020 to 2022. The emergence of D405N and N405S suggests transient evolutionary shifts, potentially representing intermediate states. Notably, S405D reappeared in 2024 within a distinct clade, indicating a possible adaptive event. High support values at key nodes reinforce the reliability of these phylogenetic relationships. As shown in Figure B, temporal analysis further confirms that D405D remained dominant from 2020 to 2022, while D405N and N405S gradually increased in prevalence, particularly in 2022. A distinct shift was observed in 2024, marked by the re-emergence of S405D, suggesting a potential selective advantage or convergent evolution (**Figure 7B**).

## Discussion

SARS-CoV-2 infects primary ACE2-negative HL-mECs, by using α_v_β_3_ integrin as an alternate receptor to ACE2 (Caccuri et al., 2021; Bugatti et al., 2022). Specifically, SARS-CoV-2 binding to α_v_β_3_ integrin occurs through an RGD (403–405: Arg-Gly-Asp) motif included in the RBD of the viral spike protein. Even if HL-mECs do not support virus replication, the presence of viral RNA and/or newly synthesized viral proteins into ECs induce the release of pro-inflammatory and pro-angiogenic molecules thus promoting an angiogenic phenotype (Caccuri et al., 2021; Bugatti et al., 2022).

It has been demonstrated that neither antibodies binding the epitope containing the RGD motif (Koehler et al., 2020) nor neutralizing antibodies evoked by BNT162b2 vaccination against the spike protein (Caccuri et al., 2021; Bugatti et al., 2022) are able to counteract virus infection on ECs, highlighting that they do not interfere with the integrins recognition site. These results suggest that the RGD motif is not accessible to antibodies and that conformational changes induced by a cofactor may favor its exposure and interaction with α_v_β_3_ integrin.

HSPGs are highly sulfated glycosaminoglycan ubiquitously distributed on cell surfaces. They are used by different viruses including herpes simplex virus (Bacsa et al., 2011), dengue virus (Chen et al., 1997), and human papillomavirus (Richards et al., 2013) for attachment and entry into host cells. It has been recently shown that HSPGs act as coreceptors also for SARS-CoV-2 infection (Clausen et al., 2020). In particular, SARS-CoV-2 spike protein interacts with HSPGs on cell surface and undergoes conformational leading to a better interaction with ACE2 (Milewska et al., 2014). In this view HSPGs may be also involved in conformational changes conducting to RGD exposure.

Here we show by SPR analysis that heparin, a structural analogue of HS, interacts with the spike protein and induces conformational changes, a *conditio sine qua non* for triggering RGD/α_v_β_3_ interaction. Moreover, we show that Heparinase III and sodium chlorate, catalyzing HSPGs degradation and undersulfation respectively, significantly inhibit integrin-mediated SARS-CoV-2 entry into ACE2-negative ECs. Interestingly, the altered bioavailability and activity of HSPGs not only impact SARS-CoV-2 entry into ECs, but also modify critical cellular signaling pathways and microenvironmental responses usually occurring after viral infection. Indeed, SARS-CoV-2 activates FAK-Src and ERK signaling pathways, which are mainly involved in α_v_β_3_-related angiogenesis (Pang et al., 2023). Interestingly, impairment of HSPGs interaction with the spike protein, by specific inhibitors, completely abrogates angiogenesis-related pathways, as previously demonstrated by using cilengitide, an α_v_β_3_ antagonist (Nader et al., 2021). HSPGs inhibitors also block SARS-CoV-2-induced release of pro-angiogenic molecules, in particular IL-8, FGF-2, Ang2, ET-1 and DPPIV, which have been previously shown to be secreted in the microenvironment of SARS-CoV-2-infected HL-mECs (Caccuri et al., 2021; Bugatti et al., 2022).

**Figure 8:**
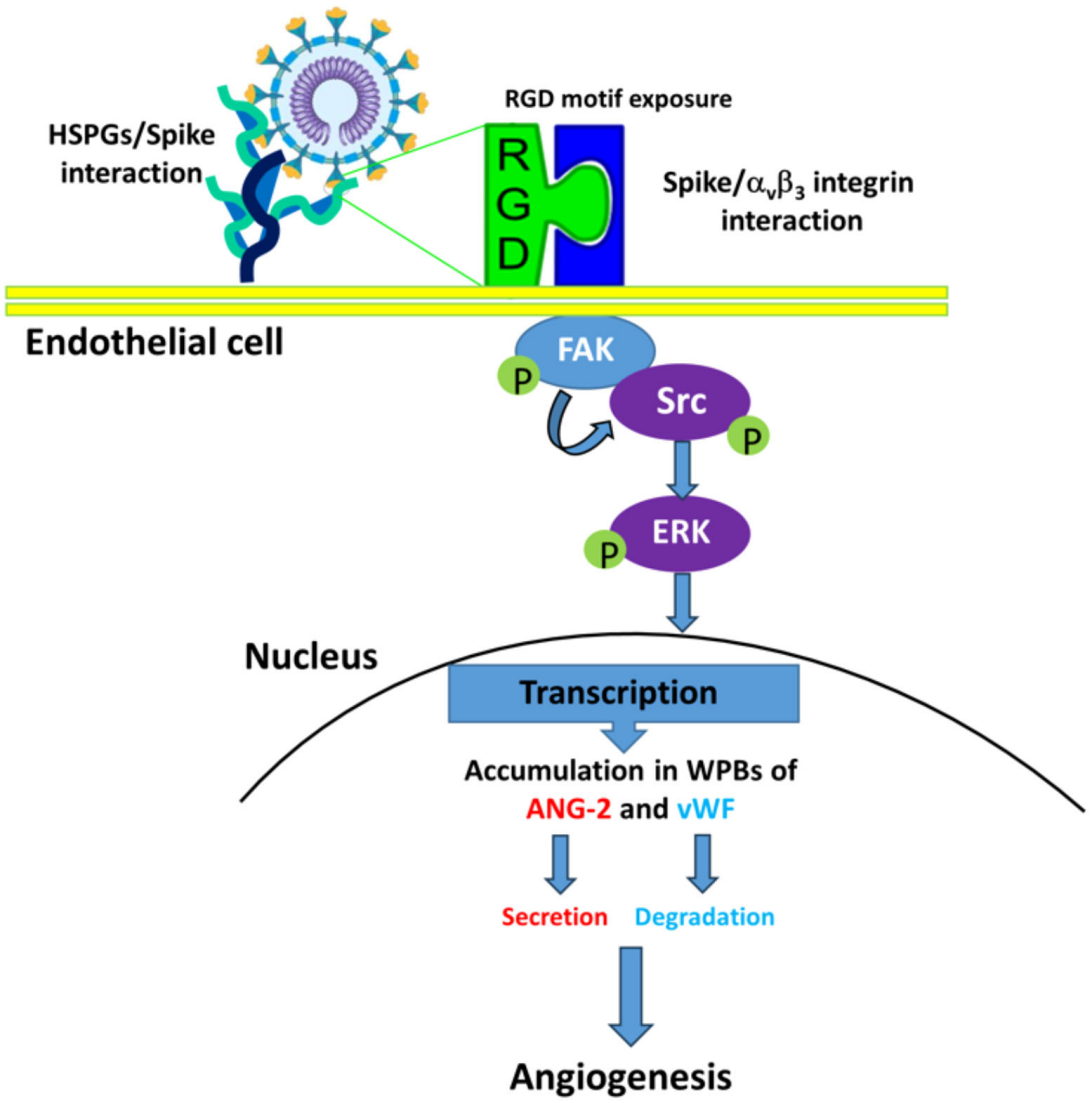
Proposed mechanisms for SARS-CoV-2-induced angiogenesis on ACE2-negative HL-mECs. HSPGs mediate spike/α_v_β_3_ interaction enhancing phosphorylation of FAK, Src and ERK, which mediates Ang-2 and vWF accumulation in WPBs of SARS-CoV-2-infected HL-mECs. Then, Ang-2 secretion and vWF degradation induces infected-HL-mECs toward an angiogenic phenotype.

α_v_β_3_ is known to play a complex role in angiogenesis, as both pro- or anti-angiogenic molecules, mostly depending on the local extracellular environment and/or the specific ligand(s) (Somanath et al., 2009). One of such ligands is vWF, a multimeric plasma glycoprotein that mediates platelet adhesion to both the subendothelial matrix and the endothelial surface. It acts as a carrier for coagulation factor VIII in the circulation and is essential for the hemostasis involved in inflammation (Casari et al., 2013). Besides its well‐characterized role in hemostasis, vWF has increasingly been implicated in angiogenic processes (Randi and Laffan, 2017). vWF regulates angiogenesis through multiple cross-talking pathways involving α_v_β_3_, VEGFR-2 and Ang2 (Starke et al., 2011) and is essential for the formation of WPBs, which contain vasoactive molecules mediating angiogenesis (Mojzisch and Brehm, 2021). Dysfunctional vWF may result in constitutive release of WPB components such as Ang2. It is known that vWF controls Ang2 levels by promoting its storage and inhibiting its synthesis (Randi et al., 2013). Indeed, inhibition of vWF induces angiogenesis on ECs and is coupled with increased release of Ang2 (Randi et al., 2018). Thus, it can be hypothesized that vWF modulates angiogenesis by regulating Ang2 storage in ECs. The latter is known to promote angiogenesis by destabilizing blood vessels and acting synergistically with VEGF-A (Randi et al., 2013). Here we demonstrate that upon SARS-CoV-2 infection, vWF accumulates within − but is not secreted by − ECs leading to the hypothesis that this factor is degraded in WPBs thus allowing Ang2 release. Taken together, our findings support the hypothesis that dysfunctional vWF metabolism coupled with Ang2 signaling accompanied to a dysregulated anti-angiogenic and inflammatory environment contribute to promoting angiogenesis during SARS-CoV-2 infection (**Figure 8**). Additional studies may lead to insight into whether targeting the vWF-Ang2 axis may prevent aberrant angiogenesis observed in the lung of SARS-CoV-2 infected patients.

SARS-CoV-2 underwent mutagenesis over time and some mutations are considered to be part of its adaptive evolution process leading to changes in disease severity and immunological responses including antigenic properties, immune evasion and sensitivity to therapeutics. The viral fitness, which influences the virus ability to infect, replicate and spread, is highly dynamic and depends on a variety of factors such as virus properties and escape of immunity. Thus, it is of extreme importance to understand the drivers of SARS-CoV-2 fitness and the prediction of the mutational pathways by which the virus will evolve. Among the multiple variations, the virus switched the RGD to RGN motif thus acquiring two loss of function, fitness to infect ECs and capability to promote EC dysfunction (Caccuri et al., 2021; Bugatti et al., 2022). In particular, this event occurred with Omicron subvariants that emerged upon time (Cao et al., 2022). Virus evolution is unpredictable, however, it is more likely that future variants will be derived from prior or contemporary VOCs, most recently exemplified by the spate of ‘second-generation’ Omicron variants derived from BA.2 (Carabelli et al., 2023). Of interest, our phylogenetic analysis revealed that after the mutation D405N/S, in recent clades appeared sequences carrying the back-mutation S405D, indicating a reversion that suggests potential selective pressures influencing these substitutions as an adaptation to environmental or host-related factors. Moreover, the fact that sequences carrying S405D were sampled across diverse regions underscores the global circulation and evolutionary adaptation of SARS-CoV-2. The homoplastic back mutation S405D brings back the ability of SARS-CoV-2 to infect ECs, allowing endothelium dysfunction, thus highlighting the need of a vigilant oversight correlated with clinical data that may be of interest for patient treatment. Tracking the emergence of SARS-CoV-2 variants as potentially more virulent or antigenically significant will help to guide the implementation of targeted control measures and further characterization.

In conclusion, HSPGs represent an attractive target molecule for potential antiviral therapeutics, since they serve as a critical player in the infection of a wide range of viruses, either directly or indirectly (Cagno et al., 2015; Rusnati and Lembo, 2016). Different studies have evaluated a variety of approaches for this purpose (i.e. nanoparticle or peptides) (Donalisio et al., 2012). Data on the interactions between the RBD and HSs, revealed several low-specificity binding modes and potential interaction sites (Parafioriti et al., 2023; Yang et al., 2024). These evidences suggest that HSPGs-targeted therapies can efficiently control SARS-CoV-2 infection being effective intervention strategies (Yang et al., 2024).

COVID-19 is frequently associated with coagulopathy and inflammation, which are clinical targets of heparin (Zhang et al., 2022). Indeed, heparin and its derivatives have been proven to be also useful anti-coagulant and anti-inflammatory molecules (Banik et al., 2021). Thus they may represent a winning therapeutic approach, to not only prevent SARS-CoV-2 infection of ECs, but also to impede endothelial dysfunctions related to viral infection.

## Data Availability

The original contributions presented in the study are included in the article/supplementary material. Further inquiries can be directed to the corresponding author.
